# Editorial: New challenges and future perspectives in cognitive neuroscience

**DOI:** 10.3389/fnhum.2024.1390788

**Published:** 2024-03-08

**Authors:** Christos A. Frantzidis, Eleni Peristeri, Maria Andreou, Alexandra I. Cristea

**Affiliations:** ^1^School of Computer Science, University of Lincoln, Lincoln, United Kingdom; ^2^Department of English Studies, Aristotle University of Thessaloniki, Thessaloniki, Greece; ^3^Department of Speech and Language Therapy, University of Peloponnese, Kalamata, Greece; ^4^Department of Computer Science, Durham University, Durham, United Kingdom

**Keywords:** cognitive neuroscience, mechanistic perspectives, aging neuroscience, memory, social neuroscience, consciousness, creativity, human-AI interaction

In recent years, we have confronted a convergence of challenges, including a global pandemic, unparalleled digital transformation, and a multitude of humanitarian challenges such as climate change, immigration, and the aging population. These crises have not only profoundly influenced our physical and mental wellbeing but have also catalyzed a revolution in the ways we engage with one another, whether in-person or through digital channels. Considering these profound changes, cognitive neuroscience assumes a pivotal role in shedding light on how external stimuli impact our brain function and mental health. This Research Topic of articles aims to explore novel challenges and future perspectives within the realm of cognitive neuroscience, at the same time offering insights into how our understanding of the brain can inform responses to the evolving landscape of crises and societal changes. Through rigorous research and interdisciplinary collaboration, this endeavor seeks to elucidate the complex interplay between external factors and our cognitive wellbeing, thereby contributing to more informed decision-making and improved outcomes for individuals and communities alike.

The transition from a phenomenological to a mechanistic perspective poses a significant hurdle for cognitive neuroscience research in the realm of mental health (Beste). While clinical practice drives the quest for identifying statistically significant variances between two population cohorts (healthy vs. pathological), this conventional method narrowly fixates on symptomatic phenomena, neglecting the exploration of underlying functional and neurobehavioral mechanisms. Moreover, it lacks a robust theoretical foundation and exhibits biases toward participants primarily sourced from Western, educated, industrialized, affluent, and democratic societies. Such biases not only exacerbate the stigma surrounding vulnerable participants but also propel cognitive neuroscientists toward embracing a “synthetic approach.” This alternative methodology pivots toward established theoretical frameworks, offering a causal comprehension of how neuroscientific mechanisms precipitate various clinical manifestations. By integrating this mechanistic approach with a strategic shift in research and by prioritizing the publication of “negative results” and the implementation of well-powered studies with appropriate statistical methodologies, researchers can yield more tangible outcomes with significant societal ramifications and clearer insights into heterogeneity.

The observational study conducted by Chatzidimitriou et al. involved a cohort of 13 patients afflicted with frontotemporal dementia (FTD). Their investigation revealed a complex interplay among executive dysfunctions, as evidenced by deficits in instrumental activities, behavioral symptomatology, such as apathy, and functional impairment. Moreover, the study underscores the pivotal role of personalized interventions in mitigating the progressive functional decline experienced by individuals with FTD.

Doheny and Lighthall's work delves into the intricacies of social cognitive neuroscience within the digital landscape, shedding light on the formidable challenges encountered in this domain. Initially, it elucidates our current understanding of the social cognitive network, drawing heavily upon the influential model proposed by Adolphs (2009). Subsequently, it delineates critical disparities in the neural underpinnings of the social brain between traditional face-to-face interactions and digital communication mediums. The discussion extends to the ramifications of this transition on higher-order social cognitive processes and its potential impact on the developmental trajectory of the brain in children and adolescents. Moreover, the study investigates how the unique context of the pandemic era and the enforced social isolation has exacerbated these challenges. It concludes by advocating for further longitudinal research to comprehensively explore the repercussions of this transition in social interaction, emphasizing the imperative of understanding its effects on both individual and societal levels.

The seminal research conducted by Ladas et al. underscores the limitations inherent in contemporary consciousness analysis, particularly concerning the reliance on sleep landmarks derived solely from oscillatory activity. Their work highlights a paradigm shift, advocating for the integration of aperiodic activity analysis during sleep alongside a mechanistic approach firmly rooted in robust philosophical foundations. Their main argument is that the highly dynamic nature of the aperiodic activity may form unique connectivity patterns for each individual, which act as a “brain fingerprint” reflecting subjective conscious experiences. This synthesis promises to catalyze a transformative leap forward in the field of cognitive neuroscience, redefining the parameters of consciousness studies.

The groundbreaking research spearheaded by Li and Fitzek illuminates the formidable hurdles encountered in pioneering novel digital communication infrastructures that leverage multi-sensory interactions within virtual or augmented reality realms. This emerging research domain seamlessly integrates cognitive neuroscience with various technological disciplines, including telecommunications, instrumentation technologies, software engineering, and artificial intelligence. However, the efficacy and capacity of these multi-sensory digital communication systems are intricately intertwined with challenges arising from compromised perceptual and cognitive processing due to both physiological and pathological aging mechanisms. This declin is primarily associated with fluctuations in specific neurotransmitters such as dopamine, serotonin, and noradrenaline. The intersection of neuroscientific insights with the visionary concept of the Metaverse presents a captivating and interdisciplinary frontier poised to elevate user experiences and revolutionize the dynamics of closed-loop human-machine interactions.

Magni et al. utilize the Bayesian brain approach to probe the impact of psychedelic hallucinations on creative cognition and cognitive flexibility. They explore the potential of immersive technology as a safe means to simulate the effects of psychedelics. The study meticulously examines the pivotal involvement of the prefrontal cortex in creative processing and divergent thinking. Moreover, the authors acknowledge a significant challenge posed by the vulnerability of the prefrontal cortex to aging processes. They highlight the potential utility of virtual reality as a prodromal marker for neurodegeneration, recognizing its implications for early detection and intervention. By bridging insights from cognitive neuroscience with innovative technology, Magni et al. offer valuable contributions to our understanding of psychedelic effects on cognition, and pave the way for novel approaches to studying age-related cognitive decline.

Sridhar et al. undertake a comprehensive exploration of the neural substrates underlying memory processes from the vantage point of cognitive human neuroscience. Their investigation delves deeply into various types of memory and elucidates the intricate mechanisms governing memory formation and consolidation. Special attention is devoted to elucidating the pivotal roles played by critical brain regions, notably the hippocampus and medial temporal lobe. Furthermore, the study examines the indispensable role of sleep in memory consolidation and explores how insights gleaned from animal studies can furnish a mechanistic framework for elucidating neurobiological mechanisms underlying human memory processes. Additionally, the paper addresses the primary challenges confronting this field and offers compelling future perspectives. By interlinking fundamental insights from cognitive neuroscience with empirical evidence, the study enriches our understanding of memory and charts a course for future research endeavors.

This Research Topic has delineated six primary challenges within the domain of cognitive neuroscience (Figure 1): (1) social interaction and isolation, (2) memory, (3) neurodegeneration, (4) consciousness, (5) novel human-machine interaction, and (6) creativity. To address these challenges effectively, a transition from a purely phenomenological approach to a mechanistic perspective is imperative. This shift promises to yield novel insights into the divergent functioning of the brain.

**Figure 1:**
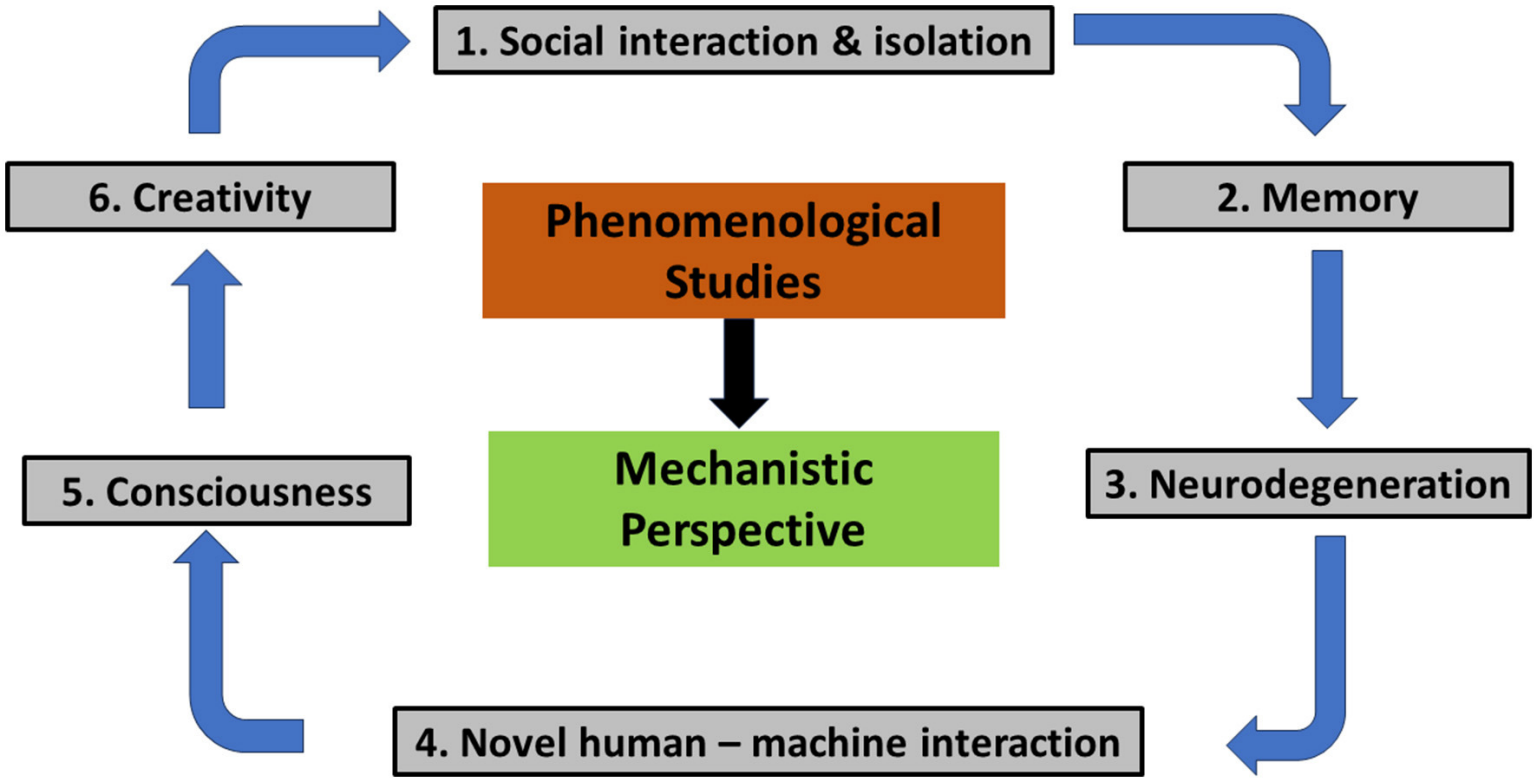
Visualization of the six (6) primary challenges within the domain of cognitive neuroscience.

As cognitive neuroscientists, it is incumbent on us to spearhead a revolution in the research agenda, aimed at destigmatizing and advocating for the needs of divergent or vulnerable populations. In pursuit of this objective, the adoption of big data acquisition and longitudinal analyses emerges as a crucial strategy. Such approaches have the potential to pivot research interests from merely identifying statistically significant differences between neurotypical and neuropathological cohorts toward a deeper comprehension of the variability in cognition and behavior. This shift aligns with the principles of precision medicine, neurodiversity and inclusiveness, ultimately fostering a more nuanced understanding of cognitive processes and promoting equitable healthcare practices.

## Author contributions

CF: Conceptualization, Methodology, Project administration, Validation, Visualization, Writing—original draft, Writing—review & editing. EP: Writing—original draft, Writing—review & editing. MA: Writing—original draft, Writing—review & editing. AC: Writing—original draft, Writing—review & editing.
